# Presence of *Plasmodium vivax* in *Anopheles gambiae* and absence in other malaria vectors in Cove-Zagnanando-Ouinhi health zone in southern Benin, West Africa

**DOI:** 10.1186/s12936-023-04834-6

**Published:** 2024-01-15

**Authors:** Tatchémè Filémon Tokponnon, Razaki Ossè, Boulais Yovogan, Ella Guidi, Constantin J. Adoha, André Sominanhouin, Juvenal Ahouandjinou, Aboubakar Sidick, Martin C. Akogbeto

**Affiliations:** 1 Centre de Recherche Entomologique de Cotonou, Ministère de la Santé, Cotonou, Benin; 2 Ecole Polytechnique d’Abomey Calavi, Université d’Abomey-Calavi, Abomey-Calavi, Benin; 3 Ecole de Gestion et d’Exploitation des Systèmes d’Elevage, Université Nationale d’Agriculture, Ketou, Benin; 4 Faculté des Sciences et Techniques, Université d’Abomey Calavi, Abomey-Calavi, Benin

**Keywords:** Malaria vectors infection, *Plasmodium* species, *P. falciparum*, *P. vivax* and *P. ovale*, COZO health zone Benin

## Abstract

**Background:**

Malaria remains a major public health problem in sub-Saharan Africa, particularly in Benin. The present study aims to evaluate the different *Plasmodium* species transmitted by malaria vectors in the communes of Cove, Zagnanado and Ouinhi, Southern Benin.

**Methods:**

The study was conducted between December 2021 and October 2022 in 60 villages spread over the three study communes. Adult mosquitoes were collected from four houses in each village using human landing catches (HLCs). After morphological identification, a subsample of *Anopheles gambiae*, *Anopheles funestus* and *Anopheles nili* was analysed by PCR to test for their infection to the different *Plasmodium* species.

**Results:**

*Anopheles gambiae* was collected at higher frequency in all the three study communes, representing 93.5% (95% CI 92.9–94) of all collected mosquitoes (n = 10,465). In total, five molecular species were found, *An. gambiae* sensu stricto (s.s.) and *Anopheles coluzzii* of the Gambiae complex, *An. funestus* and *Anopheles leesoni* of the Funestus group, and *An. nili* s.s., the sole species of the Nili group. From the five molecular species, four (*An. gambiae* s.s., *An. coluzzii*, *An. funestus* s.s. and *An. nili* s.s.) were found to be infected. *Plasmodium falciparum* was the main *Plasmodium* species in the study area, followed by *Plasmodium vivax* and *Plasmodium ovale.* Only *An. gambiae* s.s. was infected with all three *Plasmodium* species, while *An. coluzzii* was infected with two species, *P. falciparum* and *P. vivax*.

**Conclusions:**

*Plasmodium falciparum* was the only species tested for in malaria vectors in Benin, and remains the only one against which most control tools are directed. It is, therefore, necessary that particular attention be paid to secondary *Plasmodium* species for an efficient control of the disease. The presence of *P. vivax* emphasizes the need for an update of case management for malaria.

## Background

Malaria is a disease caused by a pathogen transmitted to humans by female *Anopheles* mosquitoes. Despite the goal set by the World Health Organization (WHO) to reduce the global burden of malaria to 90% by 2030 [1], malaria-related morbidity and mortality has continued to increase over the past few years. For instance, the estimated number of global malaria-related deaths was 627,000 in 2020 compared to 558,000 in 2019.

In 2019, 229 million cases of malaria were recorded worldwide, 94% of which were in the WHO regions of Africa, and 7 out of 10 malaria-related deaths were in children under 5 years of age [2]. In Benin, 46.1% of the reasons for consultation and 40.8% of hospitalization cases are due to malaria and represent the main causes of morbidity and mortality recorded especially in children under 5 years of age [3].

Malaria remains one of the most important parasitic diseases. More than 70 species of *Anopheles*, of which 30 are found in sub-Saharan Africa, have been described as vectors of *Plasmodium* [4]. Five vectors are of primary importance in Africa, including *Anopheles gambiae* and *Anopheles funestus* which are widely distributed, *Anopheles moucheti* and *Anopheles nili* which are predominantly found in forest regions, and *Anopheles mascarensis* which is present in savannah areas [5, 6].

The characterization of the species of parasites of the *Plasmodium* genus is an important step for an efficient control of malaria. A previous trial conducted in Benin showed that *Plasmodium falciparum* is transmitted by *An. gambiae* sensu lato (s.l.), *An. funestus* and *An. nili* [7, 8]. It is possible that other *Plasmodium* species may be found within these *Anopheles* vectors. In general, three methods are used for the identification of different *Plasmodium* species in different vectors. These are microscopic observation of sporozoites in dissected salivary glands [9], enzyme-linked immunosorbent assay of circumsporozoite protein (ELISA-CSP) [10] and polymerase chain reaction (PCR) [11].

Previous studies in the Ouidah-Kpomassè-Tori Bossito health zone, Southern Benin, revealed the presence of coinfections of *P. falciparum/Plasmodium malariae*, *P. falciparum/Plasmodium ovale* and *P. falciparum/P. malariae/P. ovale*) in surveyed children using microscopy [12]. In mosquitoes, a recent trial performed throughout Benin using ELISA and PCR techniques on mosquitoes found in mosquitoes of the Gambiae complex infected with both *P. falciparum* and *P. vivax* [13] in some of the 24 surveyed communes. It is, therefore, necessary to better understand the transmission dynamics of the different *Plasmodium* species in the different *Anopheles* vector species of malaria in different parts of the country. The present study was designed to determine the *Plasmodium* species infecting *An. gambiae* s.l. and other vectors in Cove, Ouinhi, and Zangnanando, three communes in COZO district Health Zone.

## Methods

### Study site

**Fig. 1:**
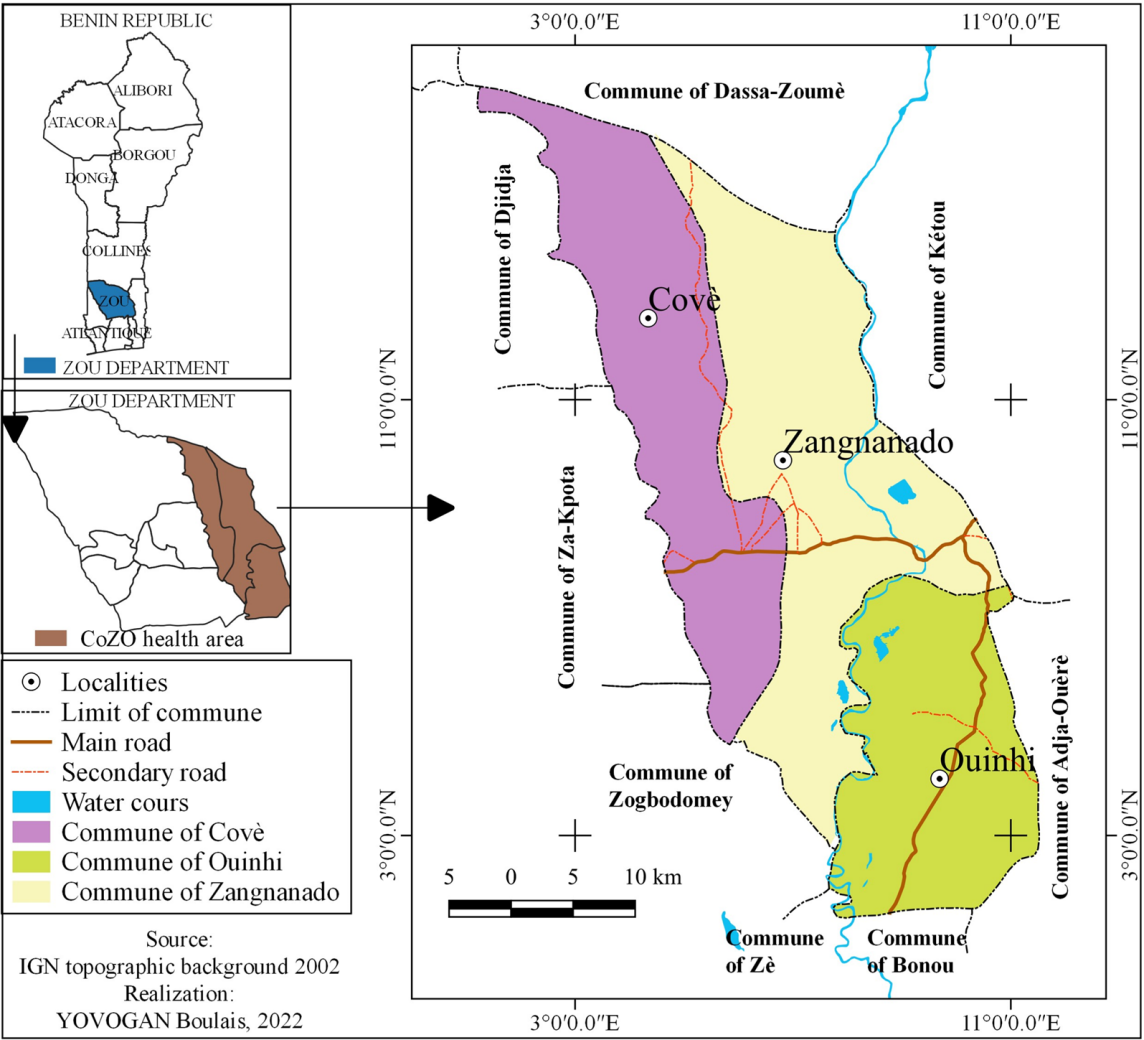
Map showing the Covè-Zangnanado-Ouinhi study area

The study took place in the communes of Cove, Zangnanado, and Ouinhi (Fig. 1) in the Zou department from December 2021 to January 2022. The Zou department covers an area of 5243 km^2^, and is bordered to the north by the Collines department, to the east by the Plateau department, to the south by the departments of Oueme and Atlantic, to the southwest by the Couffo department, and to the west by Togo. It is subdivided into nine communes namely Abomey, Agbangnizoun, Bohicon, Cove, Djidja, Ouinhi, Zangnanado, Zakpota and Zogbodomey. It is a plateau area with an altitude of 200 to 300 m. According to the fourth General Census of Population and Housing (RGPH 4) of May 2013, the department of Zou had 851,580 inhabitants. The vegetative growth period varies between 80 and 100 days. In Zou, there are two rainy seasons: (March–July and October–November) and two dry seasons (December–February and August); rainfall levels range from 1000 to 1400 mm [14].

In 2021, the annual incidence rate of malaria was 50% in children aged 0 to 4 years [15] and the prevalence of malaria infection was 36.5% in children under 5 years [16]. The communes of Cove, Zangnanado and Ouinhi are about 150 km away from Cotonou. The area has 123 villages and a population of approximately 220,000 inhabitants. The activities practiced by the population are agriculture, fishing, hunting and trade.

### Vector sampling

Mosquitoes were collected through human landing catches (HLC). In the three study communes, a total of 60 villages were randomly selected. In each village, four houses chosen at random were used for the capture of mosquitoes. Each seated collector used a flashlight as well as a sucking tube to collect the mosquitoes that landed on his/her exposed lower legs. In each house, a group of two collectors (1 indoor and 1 outdoor) worked from 19:00 to 01:00 (6 h) and the second from 01:00 to 07:00 (6 h).

### Mosquito processing

Mosquito specimens collected were morphologically identified to species level using the taxonomic identification key of Gilles and Meillon [5]. A subsample of *An. gambiae* s.l., *An. funestus* and *An. nili* randomly selected both indoors and outdoors in each commune, were separated into two parts (head–thorax, and abdomen–legs–wings) and used for molecular analyses.

### *Plasmodium* species identification by PCR

PCR was used to detect the presence of *P. falciparum*, *P. vivax*, *P. malariae*, or *P. ovale* in the *Anopheles* mosquitoes. DNA was extracted from the abdomen-legs-wings of each *Anopheles* mosquito specimen with CTAB (Cetyl-trimethyl-ammonium bromide) at 2%. After 5 min in a water bath at 65 °C, the crushed material was mixed with 200 µL of chloroform and centrifuged at 14,000 rpm for 5 min. The supernatant was gently collected in another tube containing 200 µL of isopropanol, then centrifuged at 12,000 rpm for 15 min. The supernatant was gently collected in another tube containing 200 µL of isopropanol, then centrifuged at 12,000 rpm for 15 min. The resulting pellet was then purified with 200 µL of 70% ethanol. The whole mixture was centrifuged at 14,000 rpm for 5 min. The contents of the tube were gently inverted to preserve the pellet, which was then dried for at least 3 h on the bench. Finally, 20 µL of bi-distilled water was added to the pellet, which was left in suspension on the bench overnight or for half a day. The extracted DNA was analysed by PCR according to the protocol of Padley et al. [11]. The latter allowed the identification of specific nucleotide sequences of the different *Plasmodium* species in mosquitoes.

A mixed solution was prepared which included primers specific to the *Plasmodium* species. The specific primers used to identify the different *Plasmodium* species in *An. gambiae, An. funestus* and *An. nili* were:

- *Plasmodium* (5′-AGTGTGTATCCAATCGAGTTTC-3′),
- *P. malariae* (5′-GCCCTCCAATTGCCTTCTG-3′),
- *P. falciparum* (5′-AGTTCCCCTAGAATAGTTACA-3′),
- *P. ovale* (5′-GCATAAGGAATGCAAAGAACAG-3′),
- *P. vivax* (5-AGGACTTCCAAGCCGAAGC-3′).

Positive controls consisted of *P. falciparum*, *P. ovale* and *P. malariae* DNA extracted from the blood of parasitized patients.

Amplified products for each reaction were separated using a 2% agarose gel and visualized by ethidium bromide staining. The gel images were recorded and visualized on transilluminator EBOX 1000 (Vilber, Marne-la-Vallée, France).

### Mosquito species identification through PCR

Mosquito species identification was performed on *An. gambiae* complex, *An. nili* group, and *An. funestus* group using the protocols of Santolamazza et al. [17], Kengne et al. [18], and Koekemoer et al. [19], respectively. However, the samples identified as *Anopheles* vectors were all tested to PCR following the protocol of Santolamazza et al., before being passed to the specific protocols for *Anopheles* vectors *An. funestus* and *An. nili* groups. This allows for the assessing of the involvement of each species of the *An. gambiae* complex and others species in the transmission of the different *Plasmodium* species.

### Data management and analysis

All data collected during this study were double entered in an Excel Sheet. The infection rate for each of the *Plasmodium* species identified through PCR was calculated using the following formula: Number of positive mosquitoes/Total number of mosquitoes tested × 100. Data were analysed using R Core Team software (version 4.1.3-2022) and Graph Pad Prism software version 5.0. The graphs were made using Graph Pad software version 5.0. Their confidence intervals were calculated using the exact binomial test.

## Results

### *Anopheles* species composition

Table 1 presents the *Anopheles* species composition in the study area. In total, five different *Anopheles* complexes or groups (*An*. *gambiae*, *An. funestus*, *An. nili*, *An. pharoensis* and *An. ziemanni*) were identified. *Anopheles gambiae* s.l. was collected at higher frequency in all the three study communes, representing 93.5% (95% CI 92.9–94 for the total species composition) of all collected mosquitoes (n = 10,465) (Table 1). The other malaria vector complexes and groups were in very low proportions, ranging between 0.6% (CI = 0.4–0.8) and 3.8% (CI = 3.4–4.2).

**Table 1:** *Anopheles* species composition

Species	Cove (%)	Zagnanado (%)	Ouinhi (%)	Total (%)	95% CI
*An. gambiae*	1748 (98%)	5969 (94%)	2067 (88.7%)	9784 (93.5%)	92.9–94
*An. funestus*	23 (1.3%)	78 (1.2%)	15(0.6%)	116 (1.1%)	0.9–1.3
*An. nili*	13 (0.7%)	37 (0.6%)	14 (0.6%)	64 (0.6%)	0.4–0.8
*An. pharoensis*	5 (0.3%)	207 (3.3%)	186 (8.0%)	398 (3.8%)	3.4–4.2
*An. ziemanni*	14 (0.8%)	82 (1.3%)	67 (2.9%)	163 (1.6%)	1.3–1.8
Total	1783	6351	2331	10,465	

**Fig. 2:**
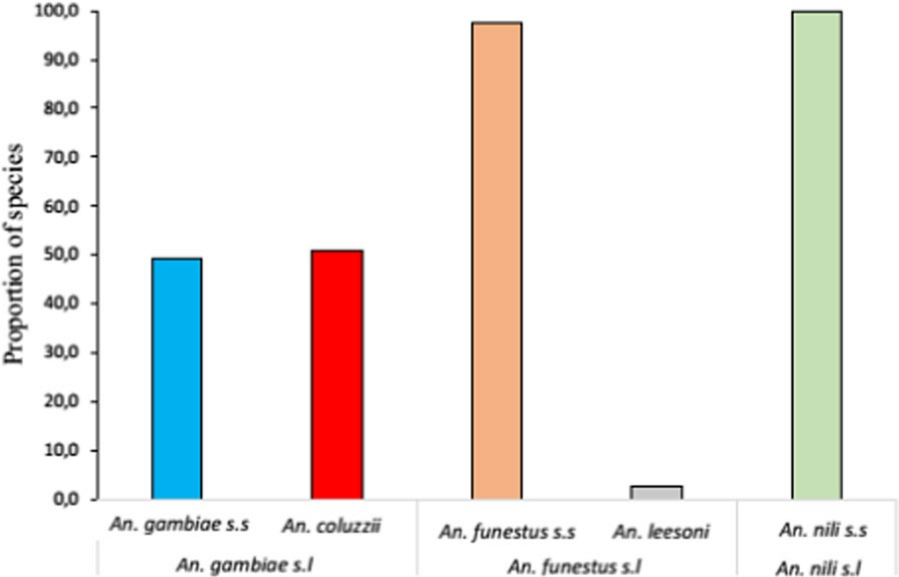
Distribution of molecular species within the *An. gambiae* complex, and the *An. funestus* and *An. nili* groups

Figure 2 shows the molecular species identified in *An. gambiae* s.l., *An. funestus* s.l. and *An. nili* s.l. Of the 338 *An. gambiae* s.l. tested in the study area, *An. gambiae* s.s. [49.1% (166/338)] and *An. coluzzii* [50.9% (172/338)] were found in similar proportion. In the *An. funestus* group, *An. funestus* s.s. was in majority (97.2%; 106/109) followed by *An. leesoni* (2.8%; 3/109). All 64 samples of the *An. nili* group were *An. nili* s.s.

### Infection rates to different *Plasmodium* species identified in the *An. gambiae* complex, and the *An. funestus* or *An. nili* groups

Combined data for all three *Anopheles* species, vector of malaria revealed mean infection rates of 8% (41/511), 3.3% (17/511), 0.4% (2/511) for *P. falciparum*, *P. vivax*, and *P*. *ovale*, respectively (Table 2).

**Table 2:** Infection rate with *P. falciparum*, *P. vivax* and *P. ovale* infection rates in the surveyed communes

Species	Municipalities	Nb tested	*P. falciparum*		*P. vivax*		*P. ovale*	
			N+	% (95% CI)	N+	% (95% CI)	N+	% (95% CI)
*An. gambiae*	Cove	89	3	3.4 (0.8–10.2)	4	4.5 (1.4–11.7)	0	0
	Zagnanado	181	21	11.6 (7.4–17.4)	12	6.6 (3.6–11.6)	2	1.1 (0.1–4.3)
	Ouinhi	68	7	10.3 (4.6–20.6)	1	1.5 (0.07-9)	0	0
	Total 1	338	31	9.2 (6.4–12.9)	17	5 (3-8.1)	2	0.6 (0.1–2.3)
*An. funestus*	Cove	22	1	4.5 (0.2–24.9)	0	0	0	0
	Zagnanado	81	8	9.9 (4.7–19)	0	0	0	0
	Ouinhi	6	0	0	0	0	0	0
	Total 2	109	9	8.3 (4.1–15.5)	0	0	0	0
*An. nili*	Cove	6	0	0	0	0	0	0
	Zagnanado	48	1	2.1 (0.1–12.5)	0	0	0	0
	Ouinhi	10	0	0	0	0	0	0
	Total 3	64	1	1.6 (0.08–9.5)	0	0	0	0
Cumul	Cove	117	4	1.1–9	4	3.4 (1.1–9)	0	0
	Zagnanado	310	30	9.7 (6.7–13.7)	12	3.8 (2.1–6.8)	2	1.1 (0.1–2.5)
	Ouinhi	84	7	8.3 (3.7–16.9)	1	1.2 (0.06–7.4)	0	0
	Total general	511	41	8 (5.9–10.8)	17	3.3 (2–5.3)	2	0.4 (0.07–1.5)

*N* number

### Proportion of molecular species identified in *An. gambiae* s.l., *An. funestus* s.l. and *An. nili* s.l.

In *An. gambiae* s.l., infection with *P. falciparum* and *P. vivax* was observed in the three communes with an average of 9.2% (95% CI 6.4–12.9) and 5% (95% CI 3–8.1). However, infection with *P. ovale* was only observed in the commune of Zagnanado (0.6%; 95% CI 0.1–2.3). In *An. funestus* group, only *P. falciparum* was detected in two communes (Cove and Zagnanado) with a mean infection rate of 8.3% (CI = 4.1–15.5). Similarly in the *An. nili* group only *P. falciparum* was detected in one commune (Zagnanado) with a mean infection rate of 1.6% (95% CI 0.08–9.5) (Table 2). No *P. malariae* infection was observed in the tested samples. No mosquitoes were found coinfected with multiple *Plasmodium* species.

### Distribution of *Plasmodium* species according to molecular species within the *An. gambiae* complex, and the *An. funestus* and *An. nili* groups

**Fig. 3:**
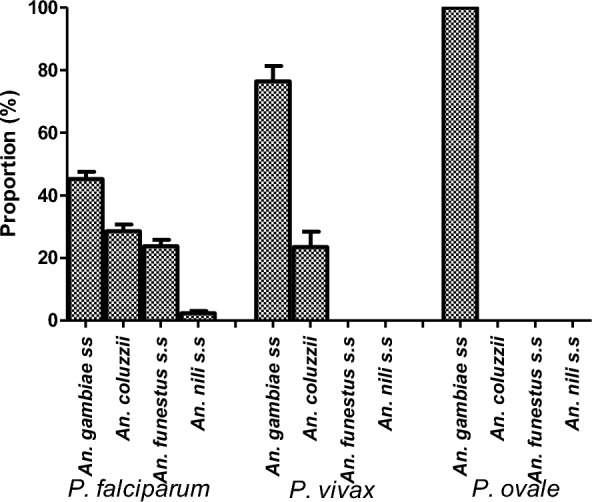
Infection rates of different *Plasmodium* species within the *An. gambiae* complex, and the *An. funestus* and *An. nili* groups

From the five molecular species identified within the three *Anopheles* complexes/groups, four forms (*An. gambiae* s.s., *An. coluzzii*, *An. funestus* s.s. and *An. nili* s.s.) were infected with *P. falciparum.* The rate of *P. falciparum* infection in *An. nili* s.s. was lower than other vectors species. Both *An. gambiae* s.s. and *An. coluzzii* were found infected with *P. vivax.* Only *An*. *gambiae* was found infected with *P. ovale* (Fig. 3).

## Discussion

The present study evaluated the presence of the infection several *Anopheles* with different *Plasmodium* species vectors in the communes of Cove, Zangnanado and Ouinhi. Overall, *P. falciparum, P ovale* and *P vivax* were found in the study area. *Plasmodium malariae* was not detected in any of the mosquitoes tested.

Species of the *An. gambiae* complex were the predominant species in the study area. This finding is consistent with previous works conducted by Ngufor et al. [20], and Yovogan et al. [21]. Molecular species identification performed in *An. gambiae* s.l. revealed the presence of *An*. *coluzzii* and *An. gambiae s.s.* in similar proportions. These results corroborate the work of Yovogan et al. [21]. The presence of *An. coluzzii* and *An. gambiae s.s.* in the study area is thought to be due to the presence of temporary larval habitats created by rainfall, and permanent and semi-permanent larval habitats created by the numerous rice paddies, as well as the tributaries of the Oueme and Zou rivers. Larvae are not generally found in moving water. It can be assumed that they are found in river beds when the water recedes during the dry season.

Within the *An. funestus* group, PCR analysis showed the presence of *An. funestus* s.s. (97.2%), and *An. leesoni* (2.8%). *Anopheles nili* s.s. was the only species identified within the *An. nili* group. These results are similar to previous works performed in some regions of Benin [8, 22–26]. They showed that while *P. falciparum* infection was reported in all three *Anopheles* complexes, but only *An*. *gambiae* s.l. was found infected with *P. vivax*, and *P. ovale*. Overall, the highest infection rate of mosquitoes was observed with *P. falciparum*, followed by *P. vivax* (3.3%) and *P. ovale* (0.4%). These results are similar to those from the work conducted in Cotonou health zones by Poirier et al. [25], and in Benin by Osse et al. [13]. Detection of *P. vivax* infection had been reported in a large-scale study in asymptomatic subjects in Benin [13, 25]. This study was the first report of the infection of *An. gambiae* s.l. with *P. ovale* in the commune of Zagnanado. The absence of infection with *P. vivax* and *P. ovale* observed in the *An. funestus* and *An. nili* groups could be due to their low density in the study area.

Infection rates for *P. vivax* in vectors were high in this area, in contrast to previous work in the area according Osse et al. [13] who found 1.5% for one commune in Agbangnizoun in the same region versus 5% in our three study communes. This is likely due to the use of PCR on whole mosquitoes for identification of parasites within the mosquitoes. This would allow for detection of different *Plasmodium* life stages within the vectors while ELISA detects only sporozoites and is usually done only on the head and thorax to avoid detection of sporozoites in the abdomens. The results from this study complement those of Osse et al. [14] expanding the geographic range of detection of different *Plasmodium* species within mosquito vectors in Benin.

Contrary to the findings of Sandeu et al. [27] no co-infection with different *Plasmodium* species was recorded in *Anopheles* mosquitoes analysed in the study. In the last study, *P. vivax* was not found in any of the mosquito samples analysed by microscopy, contrary to the molecular findings of the present study. Indeed, Howes et al. [28] have previously shown that *P. vivax* is the most widespread malaria parasite, but it is rare in Africa. African populations do not express a majority of the Duffy blood group antigens, which was thought to be essential for *P. vivax* parasite to invade red blood cells. This parasite is less the focus of public health considerations in terms of diagnosis, treatment or surveillance. As more sensitive diagnostic methods become available, *P. vivax* in Africa is increasingly reported by various types of survey (entomological, serological, community prevalence), as well as clinical data on infections of local residents and internationals [13].

The methods used in this study are not comparable to ELISAs and likely overestimated the infection rates compared to ELISAs in part due to the inherent sensitivity of PCR but also because PCR may detect *Plasmodium* life stages other than sporozoites. The use of legs, wings and abdomens for parasite detection means that non-infective stages were likely picked up [29]. However, these detections do not necessarily mean that the mosquito will transmit the parasite and constitute a potential limitation in this study. Studies have also mentioned the difficulty of ELISA in detecting the antigens of other *Plasmodium* species such as *P. malariae* and *P. ovale*, as well as the problem of antigenic variation of the CSP antigen between geographical areas [30, 31]. While PCR may overestimate sporozoite infection by *P. falciparum*, ELISAs may underestimate or even fail to detect the presence of other *Plasmodium* species.

Only *An. gambiae* s.s. was found to be infected with the three *Plasmodium* species found in the study area. In *An. coluzzii*, infection was reported with *P*. *falciparum* and *P. vivax.* Highest infection rates were observed among *An. gambiae* followed by *An. coluzzii*, *An. funestus* s.s. and *An. nili* s.s. respectively. However, the methodology used limits the conclusion that *An. gambiae* is better than *An. coluzzii* in the area. The presence of *P. vivax* in *An. coluzzii* and *An. gambiae* s.s. suggests that more detailed screening for all these vectors should be undertaken, as *P. vivax* is difficult to eliminate and more ultrasensitive methods are needed to detect infections in the community. Another limitation of this study is the non-sequencing of species in the *An. funestus* group to confirm the *An. leesoni.*

Though not yet reported from Benin, the invasive mosquito, *Anopheles stephensi*, is also a known vector of *P. vivax* and may facilitate transmission in Africa [32–34], particularly in urban settings.

## Conclusion

This study identified the presence of *P. vivax* and *P*. *ovale*, in addition to *P. falciparum* which is widespread in Benin. Molecularly, *An. gambiae* s.s.* and An. coluzzi* of the Gambiae complex, *An. funestus* s.s., and *An. leesoni* of the Funestus group, and *An. nili* s.s. of the Nili group. *Plasmodium falciparum*, *P. vivax* and *P. ovale.* were all observed to infect *An. gambiae.* Further studies are necessary to better understand the transmission dynamics of *P. vivax* in Benin.

## Data Availability

Data is contained within the article. The dataset used/or analysed during this study are available from the corresponding author on reasonable request.
